# Abstract Number ‐ 3: Large and Giant Aneurysms Outcome in the Multicenter Prospective Core Lab Adjudicated SMART Coils Registry

**DOI:** 10.1161/SVIN.03.suppl_1.003

**Published:** 2023-03-11

**Authors:** Yazan K Ashouri, Alejandro M Spiotta, Min S Park, Richard J Bellon, Bradley N Bohnstedt, Albert J Yoo, Abdallah Massad, Boris Pabon, Osama O Zaidat

**Affiliations:** ^1^ St Vincent Mercy Health Medical Center Toledo Ohio United States of America; ^2^ Department of Neurosurgery Medical University of South Carolina Charleston South Carolina United States of America; ^3^ Department of Neurosurgery University of Virginia Charlottesville Virginia United States of America; ^4^ Department of NeuroInterventional Surgery Radiology Imaging Associates Englewood Colorado United States of America; ^5^ Oklahoma University Medical Center Oklahoma City Oklahoma United States of America; ^6^ Department of Interventional Neuroradiology Texas Stroke Institute Dallas Texas United States of America; ^7^ University of Jordan Amman Jordan; ^8^ Neuroendovascular Surgery at Angioteam Medellin Colombia

## Abstract

**Introduction:**

Endovascular coiling for intracerebral aneurysms has been evolving. Yet, large, and giant aneurysms (LAGA) remain a significant challenge for treatment and carry a high rate of morbidity and mortality. Previous studies have demonstrated 72–76% long‐term adequate occlusion and up to 34% retreatment rate in patients undergoing reconstructive treatment for LAGA.

**Methods:**

The SMART registry, a prospective, multicenter outcome trial was used to identify patients with LAGA (Sac 10–24mm for large and ≥ 25mm for giant aneurysms) treated with the Penumbra SMART COIL® (SMART) system in the US and Canada. Patients’ characteristics and outcomes were then compared to patients with smaller aneurysms (SA) (Sac≤10mm). Primary effectiveness endpoint was aneurysm occlusion using Raymond‐Roy (RR) scale and safety endpoints included mortality and stroke outcomes. Fisher exact test and T‐test were used to compare categorical and continuous variables, respectively. P‐value < 0.05 was considered significant.

**Results:**

A total of 131/903 (14.5%) patients had LAGA and 42 (32.1%) of those were ruptured. Patients with LAGA were older than SA (mean age: 61.9 vs 59.4, p = 0.04). Large aneurysms were mainly saccular in 75.6% of the cases (vs 87.7% of the ≤ 10 mm ones, p < 0.001). Fusiform LAGA were 8.4% (vs 1.4% of the ≤ 10 mm ones, p < 0.001). Furthermore, they are more likely to have wide neck (69.5% vs 59.7%, p = 0.042). They are less likely to have A‐Com aneurysm (10.7% vs 28%, p < 0.001), and more likely to have basilar tip aneurysms (3.1% vs 0.8%, p = 0.044) and cavernous aneurysms (4.6% vs 0.6%, p = 0.002) compared to patients with SA. Primary coiling was the main treatment modality for both SA and LAGA (43.3% vs 43.5%, respectively, p = 1.0). However, LAGA were more likely to be treated with flow diversion in addition to coiling (6.1% vs 1.0%, p < 0.001). Packing density was significantly lower in LAGA (mean (SD) = 21.2(13.1) vs 34.1(18.3), p < 0.001). Surprisingly, long‐term aneurysm RR occlusion Class I‐II on follow‐up imaging was 82.5% in LAGA vs 91.2% in SA, p = 0.016. Retreatment rates was 11.7% in LAGA vs 6.4% in SA, p = 0.063. mRS0‐2 score was 73.6% in LAGA vs 86.4% in SA, p = 0.007. All‐cause 1‐year mortality was 9.2% in LAGA vs 4.7% in SA, p = 0.054.

**Conclusions:**

Despite challenges with treating Large and giant aneurysms, SMART coiling registry demonstrated high adequate aneurysm occlusion rate on follow‐up imaging, as well as a good independent functional outcome.

